# HMGB1 Promotes a p38MAPK Associated Non-Infectious Inflammatory Response Pathway in Human Fetal Membranes

**DOI:** 10.1371/journal.pone.0113799

**Published:** 2014-12-03

**Authors:** Sarah Bredeson, John Papaconstantinou, James H. Deford, Talar Kechichian, Tariq A. Syed, George R. Saade, Ramkumar Menon

**Affiliations:** 1 Division of Maternal-Fetal Medicine and Perinatal Research, Department of Obstetrics and Gynecology, The University of Texas Medical Branch at Galveston, Galveston, Texas, United States of America; 2 Department of Biochemistry and Molecular Biology, NHLBI Proteomics Center on Airway Inflammation and UTMB Biomolecular Resource Facility, University of Texas Medical Branch, Galveston, Texas, United States of America; University of Cincinnati, United States of America

## Abstract

**Objective:**

Spontaneous preterm birth (PTB) and preterm prelabor rupture of membranes (pPROM) are major pregnancy complications often associated with a fetal inflammatory response. Biomolecular markers of this fetal inflammatory response to both infectious and non-infectious risk factors and their contribution to PTB and pPROM mechanism are still unclear. This study examined fetal membrane production, activation and mechanistic properties of high mobility group box 1 (HMGB1) as a contributor of the non-infectious fetal inflammatory response.

**Materials and Methods:**

HMGB1 transcripts and active HMGB1 were profiled in fetal membranes and amniotic fluids collected from PTB and normal term birth. In vitro, normal term not in labor fetal membranes were exposed to lipopolysaccharide (LPS) and water soluble cigarette smoke extract (CSE). HMGB1-transcripts and its protein concentrations were documented by RT-PCR and ELISA. Recombinant HMGB1 treated membranes and media were subjected to RT-PCR for HMGB1 receptors, mitogen activated protein kinase pathway analysis, cytokine levels, and Western blot for p38MAPK.

**Results:**

HMGB1 expression and its active forms were higher in PTB and pPROM than normal term membranes and amniotic fluid samples. Both LPS and CSE enhanced HMGB1 expression and release in vitro. Fetal membrane exposure to HMGB1 resulted in increased expression of TLR2 and 4 and dose-dependent activation of p38MAPK-mediated inflammation.

**Conclusions:**

HMGB1 increase by fetal membrane cells in response to either oxidative stress or infection can provide a positive feedback loop generating non-infectious inflammatory activation. Activation of p38MAPK by HMGB1 promotes development of the senescence phenotype and senescence associated sterile inflammation. HMGB1 activity is an important regulator of the fetal inflammatory response regardless of infection.

## Introduction

Spontaneous preterm birth (PTB) and preterm prelabor rupture of the fetal membranes (pPROM) are two major pregnancy complications that are well known to be associated with intra-amniotic inflammation [1]–[3]. However, it is difficult to ascertain the exact causality and risk-predicting biomarkers of PTB and pPROM [4]. High-mobility group box 1 (HMGB1) is a highly conserved inflammatory cytokine-like alarmin that is variably expressed in many cell types [5]. The 25 kD protein was originally discovered as a nuclear protein, but has since been found to be expressed on cell surface membranes, in cytosol, mitochondria, and released into the extracellular space [6]–[8]. Therefore, HMGB1 functions vary depending on its location, as well as its post-translational modifications [9]. Intracellular HMGB1 is a non-histone chromatin-associated nuclear protein functioning as a double-stranded DNA chaperone and binding protein [10], [11] that stabilizes nucleosomes, plays a part in DNA repair and recombination, and regulates gene transcription in a non-sequence-specific fashion [3], [12], [13]. HMGB1 is typically localized in the nucleus; however, post-translational modifications, like acetylation of lysine-residues, promotes HMGB1's nuclear-cytoplasmic translocation and release from the cell [14].

Outside the cell, HMGB1 functions as a proinflammatory responder to exogenous factors (e.g., infection and stress). HMGB1 is actively released from various cells in response to oxidative stress, bacterial antigens, cytokines, or tissue injury [15], [16] and passively by necrotic cells [17]. Upon secretion, HMGB1 recruits and activates receptor-expressing cells of the innate immune system that together produce pro-inflammatory cytokines [18], [19]. HMGB1 mediates its activities through multiple receptors like the receptor for advanced glycation end products (RAGE) [20] and toll-like receptors 2 and 4 (TLR2, TLR4) [21]. The binding of HMGB1 to these receptors activates various mitogen-activated protein kinase (MAPK) pathways including the p38MAPK stress response pathway in a tissue dependent way [22], [23]. One of the consequences of cellular stress is senescence and via p38MAPK, HMGB1 may play a critical role in likely activation of senescence in PTB and pPROM.

HMGB1 is expressed by human endometrium [24], placenta [15], decidua, cervix [25], amnion epithelial cells, and in the macrophages and neutrophils during histologic chorioamnionitis [3], [11]. Different concentrations of HMGB1 in the amniotic fluid of laboring (term and preterm) and non-laboring women suggest that HMGB1 may be translocated from maternal-fetal cells and eventually released into the amniotic fluid [3]. Recent studies by Romero et al documented the significance of HMGB1 in amniotic fluid sterile inflammation [26]. To better understand the role of HMGB1 in PTB and pPROM and to characterize its functions, we investigated 1) the expression differences of HMGB1 transcripts in human fetal membranes between PTB, pPROM, and term deliveries and presence of its modified (acetylated) and secreted form in the amniotic fluid; 2) differential expression of HMGB1 in tissues exposed to PTB/pPROM risk factors, water soluble cigarette smoke extract (CSE) and lipopolysaccharide (LPS); 3) the mechanistic pathways induced by recombinant HMGB1 in human fetal membranes by examining its receptor gene expression, cell signaling pathways, and changes in inflammatory markers; and 4) the mechanistic role of HMGB1 in producing sterile inflammation by inducing fetal cell senescence.

## Materials and Methods

### 2.1 Clinical samples collection

Clinical samples were collected at Centennial Medical Center Nashville, TN and in-vitro experiment samples were collected from The University of Texas Medical Branch (UTMB), John Sealy Hospital, Galveston, TX. The study protocol was approved by the Western Institutional Review Board, Seattle, WA and Institutional Review Board at The University of Texas Medical Branch, respectively. Informed, written consent was obtained from subjects prior to sample collection. Enrollment occurred at the time of admission for delivery.

Placentas were collected from women at term (control) (n = 9), with spontaneous preterm birth and intact fetal membranes (PTB) (n = 9) and with preterm prelabor rupture of membranes (pPROM) (n = 8) after delivery. PTB samples (2 contractions/10 minutes leading to delivery with intact membranes) and pPROM confirmed by Amnisure test followed by preterm birth were included as cases. Controls were women with spontaneous labor and delivery (>37^0/7^ weeks) with no pregnancy-related complications or prior history of PTB or pPROM. Fetal membranes were dissected from the mid-zone portion, away from the placental bed and those overlaying the cervix, and stored in RNA stabilization reagent at −80°C. PTB and pPROM samples chosen for this study were matched for gestational age (<34 weeks gestation) and other clinical demographic characteristics.

### 2.2 Fetal membrane preparation and CSE/LPS/HMGB1 stimulation

Fetal membranes from the placenta of women who underwent elective repeat cesarean section deliveries at term (not in labor with an uncomplicated pregnancy) were maintained in an organ explant system as previously described [27], [28] and treated with either water soluble CSE (1∶10 dilution) (n = 11) (as detailed previously [29]), LPS (100 ng/ml in culture media; *Escherichia coli* O55:B5; Sigma, St. Louis, MO) (n = 11) (control n = 13), or recombinant HMGB1 (a gift from Professor Alexander Kurosky, PhD, Dept. of Biochemistry & Molecular Biology, UTMB) (1, 5, 10, 50 ng/ml in culture media) (n = 8) [3]. The doses were chosen based on the range of HMGB1 concentrations reported in PTB and pPROM complicated by intraamniotic infections [3]. After a pre-incubation period of 48 h at 37°C in an atmosphere of 5% CO_2_, membranes were stimulated with CSE, LPS, or HMGB1 for an additional 24 h. Tissue and media samples from stimulated and unstimulated control cultures were collected, frozen, and stored at −80°C.

### 2.3 RNA isolation, cDNA preparation, and reverse transcription PCR

Fetal membranes were disrupted with a Polytron homogenizer (Next Advanced Inc. Bullet blender, NY, USA) using 1.0-mm ZrSiO beads (Next Advanced Inc.) and Trizol reagent (Life Technologies, CA, USA). RNA was extracted using Direct-zol RNA Mini Prep (Zymo-Research, CA, USA), according to the manufacturer's instructions. RNA samples (0.1 mg/mL) were subjected to reverse transcription by High-Capacity cDNA Archive Kit (Applied Biosystems, CA, USA), in accordance with the manufacturer's instructions.

Primer-probe real-time PCR was performed using an ABI 7500 Fast Real Time PCR System (Applied Biosystems, CA). Human HMGB1, RAGE, TLR2, and TLR4 primers were obtained as pre-made TaqMan Gene Expression Assays (Life Technologies, CA). Primer specificities were tested by reverse transcription PCR (RT-PCR) and confirmed by melting (dissociation) curve analysis. GAPDH was used as an internal control. Amplification was performed under the following conditions: denaturation for 30 seconds at 95°C followed by 40 cycles of denaturation for 15 seconds at 95°C, and annealing/extension for 1 minute at 60°C. All reactions were performed in duplicate and template controls were included in each run. The comparative 2^−ΔΔCt^ method was used to calculate relative quantification of gene expression [30].

### 2.4 Acetylated HMGB1 in amniotic fluid (AF) of clinical specimens

HMGB1, a nuclear protein, is modified by acetylation prior to secretion. We tested acetylated HMGB1 in the AF of clinical specimens to confirm availability of modified and secreted (acetylated) HMGB1 in AF. Protein concentrations were quantified in AF samples (BCA Protein Assay Kit, Thermo Fisher Scientific, Waltham, MA) and AF volume corresponding to equal total protein concentrations were fractionated by a modified two-dimensional HPLC system (Beckman Coulter Biomek 2000 robot, Fullerton, California). This system resolves complex protein mixtures by anion exchange chromatofocusing in the first dimension and hydrophobicity (reverse phase chromatography) in the second dimension. Fractions were separated in the first dimension column by pH, re-injected into the second dimension reverse phase column, and then separated by hydrophobicity. The separated fractions were printed on PVDF membrane using a modified Schleicher & Schuell dot blotting apparatus. PVDF membranes were probed for HMGB1 and acetylated lysine (Abcam, Cambridge, MA) by Western blot analysis. As acetylation can be inhibited by glucocorticoids, a stratified analysis of our data was performed to confirm that the modification (acetylation) of HMGB1 is not a function of steroid use during pregnancy complications.

### 2.5 HMGB1 and cytokine assays

HMGB1 concentration in the supernatants of CSE, LPS-treated, and untreated control fetal membrane culture media was further tested by a Human HMGB1 Competitive Enzyme Immunoassay (ELISA, MyBioSource, CA, USA), per manufacturer's instructions, where intensity of color detected was inversely proportional to the HMGB1 concentration. Of note, this assay does not discriminate between acetylated and non-acetylated HMGB1 and therefore indicates overall changes in HMGB1.

Cytokine measurement in HMGB1-treated and untreated control culture media was performed using a customized Human Cytokine Panel (EMD Millipore, MA, USA) for the PTB and pPROM cytokines IL1β, TNFα, and IL-6, and was detected by Luminex xMAP technology (Luminex, TX, USA).

### 2.6 MAPK panel assay

To document the signal transductions induced by HMGB1 treatment of amniochorion, 9-panel phosphorylated protein measurements were performed in tissue homogenates. The degree of phosphorylation for each analyte was quantified using Human MAPK/SAPK Cell Signaling Multiplex Assay (EMD Millipore, MA, USA), which detects the following phosphoprotein targets: ATF-2 (Thr71), Erk1/2 (Thr185/Tyr187), HSP27 (Ser78), JNK (Thr183/Tyr185), MSK1 (Ser212), MEK1 (Ser222), p38MAPK (Thr180/Tyr182), p53 (Ser15), c-Jun (Ser73), Stat1 (Tyr707). Briefly, 25 µg of freshly homogenized tissue was incubated overnight with premixed beads coated with specific capture antibodies that are directed against the desired biomarkers in a 96-well plate. Manufacturer's instructions were followed. The plate was read on the Luminex LX 200 using the Xponent Software. Data was interpreted as a qualitative analysis with the value of each analyte. Verification of the assay was done using positive and negative cell lysate controls provided by the manufacturer.

### 2.7 Senescence by senescence-associated β -galactosidase assay (SA-β-gal)

Primary amnion epithelial cells were used to demonstrate activation of SA-β-gal induction in response to HMGB1. Primary amnion cells (cultured from normal term not in labor placental membranes) were treated with 50 ng/ml of HMGB1. The expression of the SA-β-gal biomarker is independent of DNA synthesis and distinguishes senescent from quiescent cells [31]. This enzymatic activity is distinct from the ubiquitous acidic β-galactosidase and can be detected at pH 6.0 with the chromogenic substrate X-gal. Senescent cells were identified using a histochemical staining kit (Sigma, St. Louis, MO) with blue cells visualized by light microscopy 3 h after treatment with HMGB1. The proportion of positive cells in the total cell population was counted manually and reported for HMGB1-treated and untreated cultures.

### 2.8 NF-κB activation

To document the dependency of transcriptional activator NF-κB in cytokine activation after HMGB1 treatment and p38MAPK activation, we tested the phosphorylation of NF-κB/RelA subunit (phosphorylation of ser 276). Proteins extracted from tissues (n = 3) treated with HMGB1 (50 ng/ml) and HMGB1 alone or HMGB1 and p38MAPK inhibitor (SB203580) were subjected to Western blot analysis. SB203580 (30 µM) ((4-(4-fluorophenyl)-2-(4-methylsulfinylphenyl)-5-(4-pyridyl)1H-imidazole), p38MAPK inhibitor [32], [33] (Cell Signaling #5633, Danvers, MA) was used to confirm p38MAPK effect on Rel A phosphorylation.

### 2.9 Statistical analysis

All data were estimated using the GraphPad Prism Program version 6.0 (GraphPad, San Diego, CA, USA). Data shown in figures were expressed as the mean ± standard error of the mean (SEM) and homogeneity of data was assessed by a Kolmogorov-Smirnov normality tests. All data was analyzed by one-way ANOVA followed by Tukey's post hoc test. Non-parametric Mann-Whitney tests were performed wherever necessary and data were significant at p<0.05.

## Results

### 3.1 Expression of HMGB1 mRNA in fetal membranes from adverse pregnancy outcomes (PTB and pPROM)

**Figure 1:**
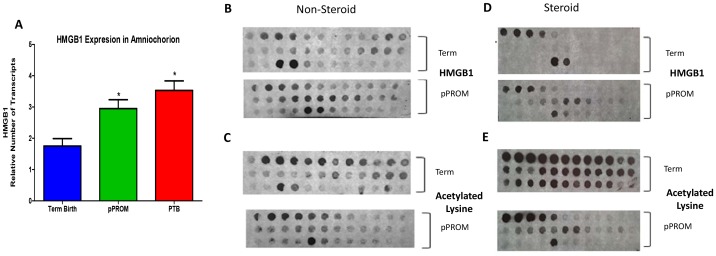
HMGB1 Expression in Fetal Membrane and Amniotic Fluid Cases and Controls A. Bar graph shows HMGB1 expression in fetal membranes as the mean and standard error of relative number of transcripts as determined by real time PCR. GAPDH was used as an internal control. Comparison among the clinical samples from women at term not in labor (Term Birth), with preterm premature rupture of membranes (pPROM), and with preterm birth with intact membranes (PTB). (ANOVA, *p<0.05). **B-E.** HMGB1 and acetylated lysine in human amniotic fluid (AF). **B.** pPROM AF without steroids has a greater amount of HMGB1 than term AF. **C**. Term AF has a greater amount of total protein acetylation than pPROM AF without steroids. **D**. pPROM AF with glucocorticoids has a greater amount of HMGB1 than term AF. **E**. Term AF has a greater amount of total protein acetylation than pPROM AF with steroids. However, HMGB1 retains acetylation in pPROM despite deacetylation of proteins by administered steroids (*D vs. E*) as seen by the preserved HMGB1 pattern. Interestingly, more proteins are acetylated in pPROM AF without steroid use than with steroid administration (*C vs. E*). In both term and pPROM, HMGB1 shows a consistent expression pattern (*B vs. D*).

Using real time PCR, we determined the differences in HMGB1 gene expression in preterm and term fetal membranes and those with pPROM. HMGB1 expression was detected in all fetal membrane samples regardless of their status; however, both cases had higher HMGB1 expression than term membranes. PTB displayed a 2-fold difference (p = 0.0003) and pPROM exhibited a 1.7-fold (p = 0.005) increase (Figure 1A) in the number of transcripts than controls. No significant difference was seen between PTB and pPROM (p = 0.181). Table 1 lists the demographic/clinical data comparisons.

**Table 1:** Demographic and gestational characteristics of studied patients.^46^

Characteristic	TERM (n = 8)	PTB (n = 7)	pPROM (n = 8)	p
**Maternal age (y)^1^**	32±6	29±5	27±8	0.35
**Marital status ^2^**				
** Single**	3 (37.5)	0	3 (50.0)	0.14
** Married**	5 (62.5)	7 (100.0)	5 (62.5)	
**Ethnicity^2^**				
** White**	5 (62.5)	5 (83.3)	5 (62.5)	0.53
** Black**	3 (37.5)	1 (16.7)	3 (37.5)	
**Smoked during pregnancy**	0	1 (14.3)	0	0.30
**Gestational age at birth (weeks/days)^ 1^**	39±1	33±3	32±4	<0.0001
**Chorioamnionitis**	0	0	2 (25.0)	0.12
**Antenatal steroids**				
** Yes**	0	6	7	0.5
** No**	8	2	1	
**Gravidity^2^**				
** Primiparous**	1	3	3	0.38
** Multiparous**	7	4	5	

1. Anova (Tukey's multiple comparisons test).

2. Fisher' s exact test.

### 3.2 HMGB1 is secreted as acetylated proteins in the AF

For this study we used only term and pPROM amniotic fluid samples to demonstrate the presence of modified HMGB1 in both term and pPROM amniotic fluid samples (Figure 1B; D). Acetylated HMGB1 (as documented in the same blot after re-probing for acetylated lysine) was consistently present in term and pPROM AF (Figure 1C; E). Similar data were obtained from AF samples from PTB with intact membranes (data not shown). Modification of HMGB1 through acetylation allows for its secretion from fetal membranes into AF, making it a pro-inflammatory cytokine. We stratified our analysis based on data derived from steroid administration of PTB and pPROM subjects. Interestingly, HMGB1 retained acetylation status despite deacetylation of other proteins by steroids suggesting a major role by HMGB1 in PTB and pPROM inflammation (Figure 1B–E).

Remaining data were generated using in vitro fetal membrane cultures. These experiments were conducted to document the inducibility of HMGB1 expression in fetal membranes, its receptor dependency and mechanistic signaling pathways generated as a pro-inflammatory cytokine.

### 3.3 HMGB1 is increased in vitro in fetal membranes treated with PTB and pPROM proinflammatory agonists

**Figure 2:**
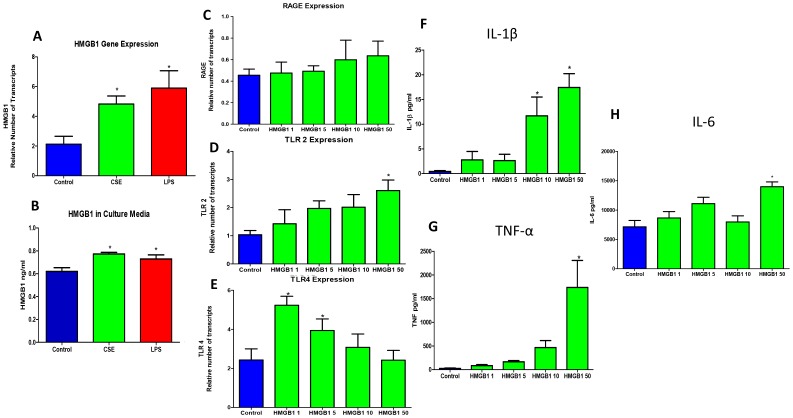
HMGB1, Receptor, and Proinflammatory Cytokine Expression in Fetal Membranes. Bar graphs show **A.** HMGB1 expression as the mean and standard error of relative number of transcripts as determined by real time PCR. GAPDH was used as an internal control. Comparison among control, cigarette smoke extract-stimulated (CSE), and lipopolysaccharide-stimulated (LPS) fetal membranes at term. **B.** HMGB1 concentration as the mean and standard error of relative intensity as determined by competitive enzyme immunoassay ELISA. Note: Intensity of color detected is inversely proportional to the HMGB1 concentration. Comparison among control, cigarette smoke extract-stimulated (CSE), and lipopolysaccharide-stimulated (LPS) culture media at term. **C–E.** HMGB1 receptor expression in HMGB1-stimulated (1, 5, 10, 50 ng/ml) fetal membranes as the mean and standard error of relative number of transcripts as determined by real time PCR. GAPDH was used as an internal control. **C.** Comparison of RAGE expression among HMGB1 treated fetal membrane samples. **D.** Comparison of TLR2 expression among HMGB1 treated fetal membrane samples. **E.** Comparison of TLR4 expression among HMGB1 treated fetal membrane samples. **F–H.** Cytokine concentration in HMGB1-stimulated (1, 5, 10, 50 ng/ml) fetal membrane culture media as the mean and standard error of relative concentration as determined by multiplex human cytokine panel analysis. **F.** Comparison of IL-1β concentration among HMGB1 treated culture media samples. **G.** Comparison of TNFα concentration among HMGB1 treated culture media samples. **H.** Comparison of IL-6 concentration among HMGB1 treated culture media samples. (ANOVA, *p<0.05).

In order to document differential expression and secretion of HMGB1 in response to two distinct PTB and pPROM risk factors, we treated tissues with CSE and LPS to generate oxidative stress and inflammation respectively. All tissue samples (treated and untreated controls) produced measurable quantities of HMGB1 (Figure 2A). CSE-treated tissues responded with a 2.3-fold HMGB1 increase (p = 0.031), while LPS treatment showed a 2.8-fold HMGB1 increase (p = 0.035) compared to untreated controls. No significant difference was seen between CSE and LPS treated samples (p = 0.22). Increased expression seen in tissues were reflected in the media after stimulation with CSE showing a 1.4-fold increase in HMGB1 concentration (p = 0.004) and LPS with a 1.3-fold HMGB1 concentration increase (p = 0.015) compared to untreated controls (Figure 2B). HMGB1 is secreted from cells to perform autocrine functions in response to oxidative stress and inflammation. HMGB1 protein in culture media was not different between CSE and LPS (p = 0.39).

### 3.4 HMGB1 induces receptor expression in fetal membranes

Secreted HMGB1 influences cells through autocrine and paracrine fashion by inducing its own receptors; namely RAGE, TLR2, and TLR4. To further characterize HMGB1 mediated activity, real time PCR was used to document dose dependent changes in the expression of HMGB1 receptors in fetal membranes. Expression of RAGE was not different with different doses of HMGB1 (1, 5, 10, and 50 ng/ml) compared to untreated controls (Figure 2C); however, TLR2 and TLR4 expression patterns differed in response to HMGB1. TLR2 expression increased dose-dependently reaching significance with 50 ng/ml HMGB1 treatment (p = 0.009) (Figure 2D). Conversely, TLR4 expression decreased dose-dependently with significantly more TLR4 expression seen in 1 ng/ml (p = 0.004) and with marginal significance at 5 ng/ml HMGB1-stimulation (p = 0.06) (Figure 2E).

### 3.5 HMGB1 induces Pp38 MAPK pathway

**Figure 3:**
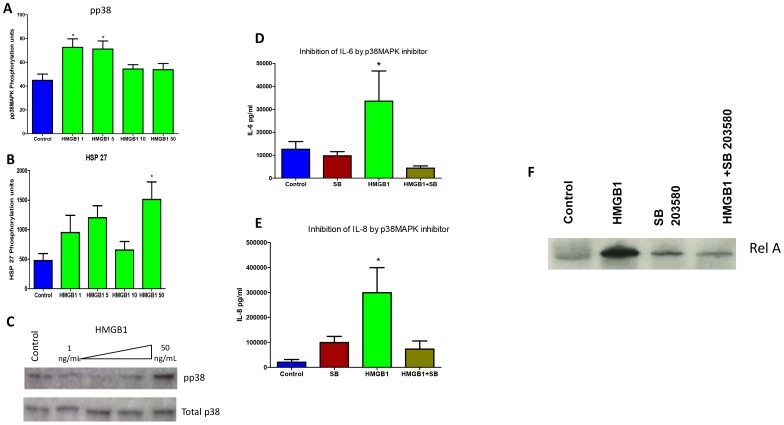
Pp38 MAPK and NF-κB Pathways Activation by HMGB1. **A–B**. Bar graphs show MAP kinase protein concentration in HMGB1-stimulated (1, 5, 10, 50 ng/mL) fetal membranes as the mean and standard error of relative intensity as determined by multiplex human MAPK protein panel analysis. **A.** Comparison of phosphorylated-p38 (Pp38) concentration among HMGB1-treated fetal membrane samples. **B.** Comparison of HSP27 concentration among HMGB1-treated fetal membrane samples. **C.** Dose dependent activation of Pp38MAPK after HMGB1 treatment where maximum activation was seen after 50 ng/ml HMGB1 treatment. **D–E.** Bar graphs show cytokine concentrations in HMGB1 (50 ng/ml) and HMGB1+SB 203580 (30 uM) p38MAPK inhibitor treated fetal membrane culture media. **D.** Comparison of IL-6 concentration among control, SB 203580 alone, HMGB1alone, and HMGB1+SB203580 cultures. **E.** Comparison of IL-8 concentration among control, SB 203580 alone, HMGB1alone, and HMGB1+SB203580 cultures. **F.** p38MAPK dependent NF-κB activation. HMGB1 (50 ng/ml) increased RelA phosphorylation. (ANOVA, *p<0.05).

To better understand the MAPK pathway activation by HMGB1, a semi quantitative assessment of phosphorylated (P) MAPK proteins was carried out. HMGB1-stimulated tissues showed activation of Pp38MAPK in tissues treated with all doses of HMGB1 (all p<0.05) (Figure 3A). Heat shock protein (HSP) 27, a downstream target of Pp38MAPK activation, was also increased after 50 ng/ml of HMGB1 (1513±785.5 U/ml) compared to controls (477.9±289.4; p = 0.02) but not at lower concentrations (Figure 3B). None of the other tested MAPK signaling molecules (ERK, JNK, STAT1, c-JUN, p53, MEK-1, MSK-1, and ATF-2) showed any significant differences. It has to be noted that ERK showed an inverse dose-dependent trend similar to that seen with TLR4. Activation of Pp38MAPK was further confirmed by Western blot analysis (Figure 3C) where a dose-dependent effect was seen. Highest level of activation was seen after 50 ng/ml HMGB1 treatment and no differences were seen with other doses.

### 3.6 HMGB1 activates amnion cell senescence phenotype

**Figure 4:**
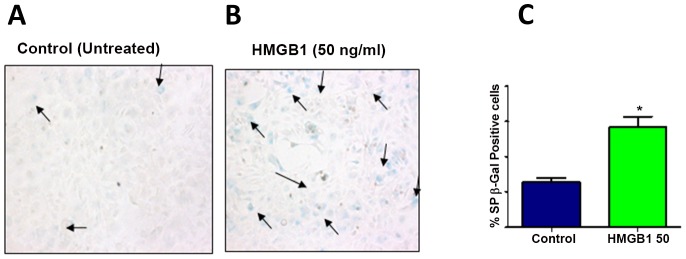
SA-β-Gal staining. **A–B.** Pp38 MAPK mediated senescence phenotype (SP) development was determined using Senescence Associated β -Galactosidase (SA β-Gal) assay. SP was seen as blue staining cells and the number of these cells were higher after 50 ng/ml of HMGB1 treatment (**B**) compared to control (**A**). **C.** Bar graph on the right shows percentage of SA β-Gal staining cells after 50 ng/ml of HMGB1 treatment compared to untreated control.

HMGB1 mediated senescence phenotype (SP) development was determined using SA β-Gal assay. SP was seen as blue staining cells and the number of these cells was higher after 50 ng/ml of HMGB1 treatment (Figure 4B) compared to control (Figure 4A). As shown in the bar graph (Figure 4C), the percentage of SA β-Gal staining cells was significantly higher after 50 ng/ml of HMGB1 treatment compared to untreated control (p<0.05).

### 3.7 HMGB1 stimulates pro-inflammatory response in human fetal membranes

To test the pro-inflammatory properties of HMGB1, we quantitated concentrations of inflammatory cytokines after HMGB1 treatment of human fetal membranes. HMGB1-treated tissues showed an increase in IL-1β with 10 ng/ml (11.6±8.5 pg/ml) and 50 ng/ml (17.4±7.3 pg/ml) of HMGB1 compared to controls (0.4±0.4 pg/ml; p = 0.0009 and 0.01 respectively; Figure 2F). Similarly, higher doses of HMGB1 substantially increased TNFα (1736±1.6 pg/ml vs. 26.4± 23.4 pg/ml; p = 0.0003; Figure 2G) and IL-6 (25.8 ±24.7 ng/ml vs. 10.1±6.9 ng/ml; p = 0.0002; Figure 2H) concentrations from fetal membranes compared to untreated controls. However, lower concentrations of HMGB1 (10, 5 and 1 ng/ml) did not produce statistically significant changes in any of these cytokine concentrations.

### 3.8 Confirmation of senescence associated secretory protein (SASP) production

After obtaining the data on Pp38 MAPK activation and cytokines (especially IL-6 and IL-8 that are also senescence related [34], [35], we verified the association between the production of these cytokines and senescence development in fetal membranes. Five separate cultures were setup to document the effect of senescence phenotype (SP) development. Fetal membranes were treated either simultaneously or independently with SB 203580 (p38MAPK inhibitor) along with 50 ng/ml of HMGB1 that produced Pp38MAPK response. After 24 h of treatment, media samples were analyzed for IL-6 and IL-8. As shown in Figures 3D and E respectively, IL-6 (299±225 ng/ml) and IL-8 (33.5±29.4 ng/ml) levels were increased after 50 ng/ml HMGB1 treatment compared to controls (20.2±25.1 and 12.6±7.6 ng/ml, respectively; both p<0.05). This HMGB1-induced increase was significantly reduced after SB203580 treatment (IL-6 4.3±2.2; p = 0.007; IL-8 73.2±2.6 ng/ml; p = 0.031) suggesting that HMGB1 exerts its effects by promoting senescence phenotype and senescence associated secretory phenotype. Treatment with p38MAPK inhibitor SB203580 alone had no effect on fetal membrane cytokine production, as the levels were similar to that of controls.

## Discussion

Little has been reported on HMGB1's role in PTB and pPROM, although a prominent role in inflammation is indicated by its increased presence in the amniotic fluid in pregnancies complicated by intraamniotic infection and inflammation regardless of the membrane status (i.e. preterm prelabor rupture of the membranes or intact membranes) [3]. Our study demonstrates that HMGB1 produced by fetal membranes causes autocrine activation of p38MAPK-mediated senescence-associated inflammation independent of infection. The key findings from this study are: 1) HMGB1 mRNA expression is higher in fetal tissues from PTB and pPROM than at normal term. 2) HMGB1 is acetylated prior to its release, which prevents its nuclear reentry and allows packaging into secretory lysosomes. Our observation that levels of acetylated HMGB1 are higher in AF samples in both PTB and pPROM suggests the presence of biologically active HMGB1 in adverse pregnancy outcomes [26]. 3) In vitro, proxies for two major risk factors of PTB and pPROM, cigarette smoking and infection, increased HMGB1 expression and its release from human fetal membranes. 4) HMGB1 treatment induced a dose dependent expression of its receptors. Expression of RAGE, a known HMGB1 receptor is not changed in fetal membranes whereas TLR2 is increased at higher doses and TLR4 at lower doses. 5) Specificity of HMGB1 is suggested by our observation that it does not induce phosphorylated ERK or JNK pathways but causes p38MAPK activation; 6) Higher doses of HMGB1 increase expression of proinflammatory cytokines (IL-1β, IL-6 IL-8 and TNFα) known to be associated with PTB and pPROM. 7) Inhibition of p38MAPK reduced both IL-6 and IL-8 release from fetal membranes.

HMGB1 is an abundant nuclear, non-histone protein, which normally functions as a facilitator of DNA transcription [36]. Two homologous High Mobility Group (HMG) Box domains provide flexibility to this protein for performing basic intranuclear functions [37], [38]. As an alarmin, HMGB1 can translocate to the cytoplasm upon cellular activation in response to oxidative stress damage to DNA or in response to other cellular injuries. Cytoplasmic HMGB1 is further modified and escorted out of the cell where it acts as an inflammatory cytokine [39]. As a cytokine, HMGB1 is pleiotropic in its functions and causes inflammation-associated pathologies [39], [40]. Of note, HMGB1's function is dependent on tissue type, location (intracellular [nuclear vs. cytoplasmic] and extracellular), post translational modifications and the receptor type utilized for signal transduction [39].

In our study, we noted higher concentrations of post-translationally modified HMGB1 in the AF from PTB and pPROM [3]. Acetylated HMGB1 was also seen in fetal membranes during these conditions (data not shown) suggesting the availability of both endogenous and exogenous pools of active HMGB1 during pregnancy complications. Acetylated HMGB1 is reported to cause its own release, and this autocrine function is expected to enhance its pro-inflammatory properties. The effect of HMGB1 on fetal membranes is dose dependent and the senescence activation (as shown by SA β-Gal staining of amnion cells after HMGB1 treatment) may occur in fetal membranes either through TLR2 (at higher doses of HMGB1) or TLR4 (at lower doses and may be with higher affinity). In vivo, in cases with intraamniotic infections, various antigens like peptidoglycan polysaccharide (PGPS) and LPS can induce expression of TLR2, TLR4 and RAGE making these receptors available for HMGB1 binding. Therefore, a synergy between various antigenic factors and HMGB1 may enhance risk. Such synergy of HMGB1 is reported in other systems [40]. A synergy between LPS and HMGB1 is reported to cause p38MAPK activation [41] through TLR4-NF-κB pathway. It is important to note that HMGB1 alone is capable of eliciting a response that leads to p38MAPK activation supporting a non–infectious inflammatory status in fetal tissues. This is critical in certain subsets of PTB and pPROM where infection is not the primary cause and in cases with sterile inflammation where the true causality is unclear [26].

**Figure 5:**
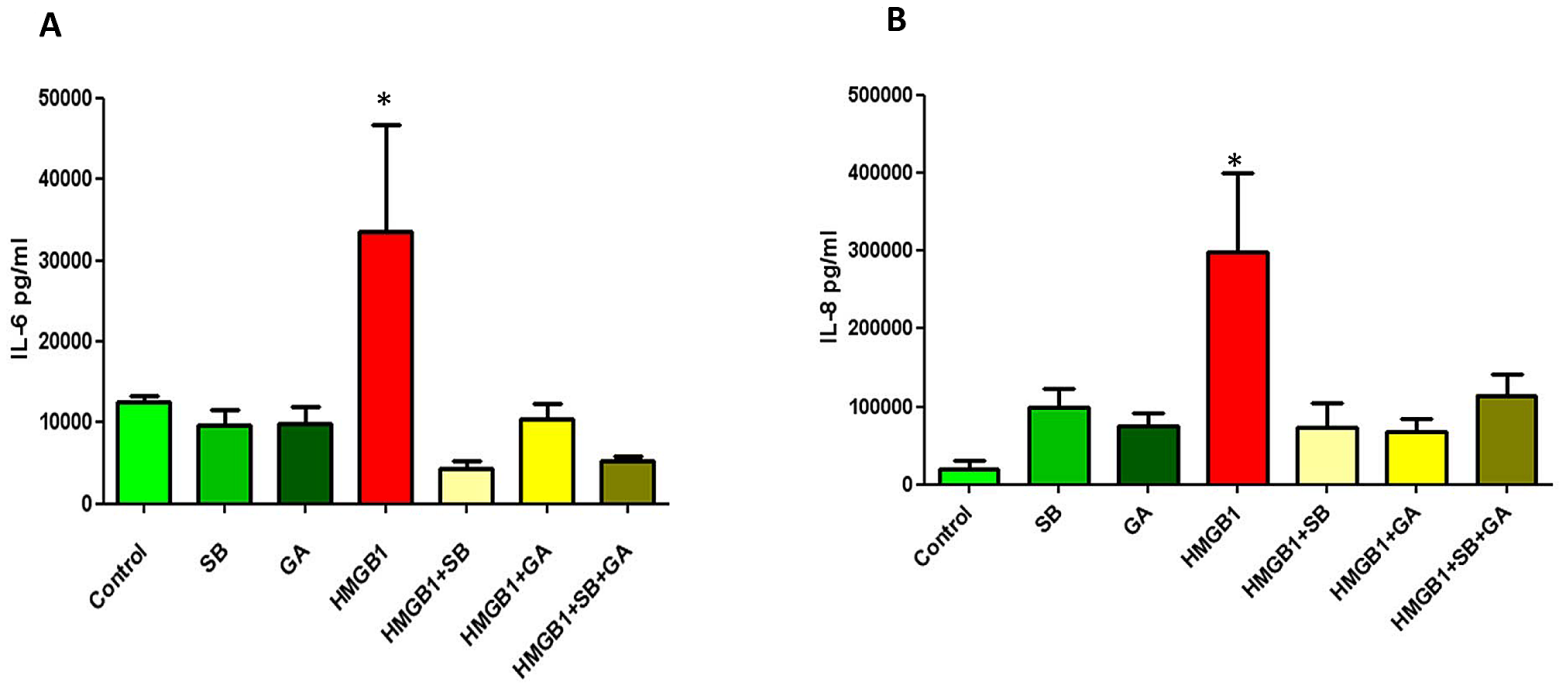
HMGB1 SASP Cytokine Production. Bar graphs show cytokine concentrations in p38MAPK inhibitor SB203580, HMGB1 inhibitor (GA), and HMGB1treated, and simultaneous HMGB1 with SB203580 (30 uM), GA (100 uM), or a combination of both SB203580 and GA treated fetal membrane culture media. **A.** Comparison of IL-6 concentration among control, SB 203580 alone, GA alone, HMGB1alone, HMGB1+SB203580, HMGB1+GA, and HMGB1+SB+GA cultures. **B.** Comparison of IL-8 concentration among control, SB 203580 alone, GA alone, HMGB1alone, HMGB1+SB203580, HMGB1+GA, and HMGB1+SB+GA cultures. (ANOVA; *p <0.05).

p38MAPK activation promotes cell cycle arrest and cellular senescence by targeting the expression of proteins of senescence phenotype. We have previously reported that chronic stressors can cause p38MAPK activation in fetal membranes, develop a senescence phenotype with biomolecular and histologic changes and create an inflammatory milieu that resembles infection. We have senescence activation by p38MAPK in amniocytes in response to cigarette smoke extract (OS inducer) and have also reported of DNA damage and existence base excision repair mechanism that can also contribute to senescence activation [44]–[46]. We postulate that the inflammatory signature observed in response to HMGB1 is due to the generation of senescence-associated secretory phenotype (SASP). Inhibition of inflammatory markers by p38MAPK inhibitors supports this hypothesis and also supports the concept of non-infectious inflammatory status. We also verified that the master transcriptional activator, NF-κB, is associated with (or involved) in the HMGB1-induced SASP activation. RelA phosphorylation was increased after treatment with 50 ng/ml of HMGB1 and was minimized to levels similar to control when inhibitors to HMGB1 (glycyrrhizic acid 10 mM/ml) and SB203580 (p38MAPK inhibitor) were used (Figure 3F). To confirm HMGB1's effect on SASP cytokine production, we further examined the response of IL-6 and IL-8 (Figure 5A; B) to HMGB1, p38MAPK inhibitor SB203580, and HMGB1 inhibitor Glycyrrhizic acid (GA) [42], [43]. We used 100uM of GA (Sigma Aldrich; Cat # 50531, St. Louis, MO). Simultaneous treatment of HMGB1 with SB203580, GA, or a combination of both SB203580 and GA, reduced both IL-6 and IL-8 concentrations to significantly less than that of HMGB1 alone (both p<0.05). These data, again, indicate HMGB1 down regulates cytokine production via p38MAPK and that down regulation of p38MAPK inhibits both NF-κB activation and SASP production.

**Figure 6:**
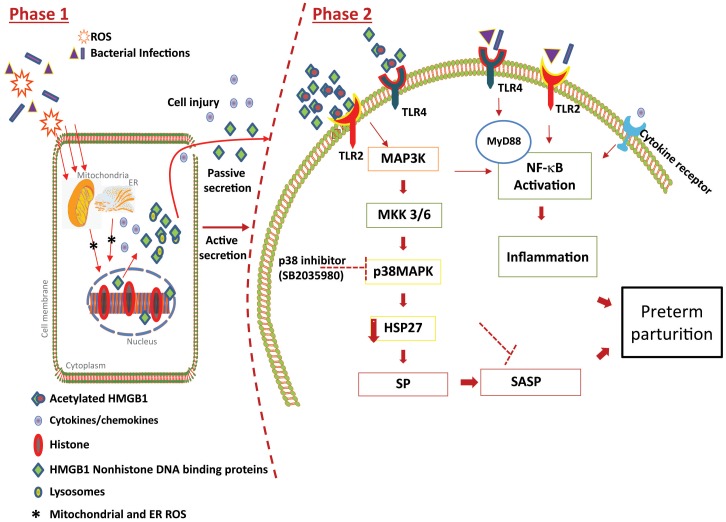
Model of HMGB1 Mediated Inflammatory Response. Proposed mechanistic pathway of HMGB1-mediated signaling in human fetal cells. In **Phase 1**, infectious or non-infectious risk factors associated with spontaneous preterm birth and preterm premature rupture of the membranes cause reactive oxygen species (ROS) formation from mitochondrial and non-mitochondrial origins in fetal membrane cells (44). This ROS causes nuclear HMGB1 to translocate into the cytoplasm. In the cytoplasm, HMGB1 is acetylated or modified by lysosomes and secreted either due to cellular injury (necrosis) or through carriers. HMGB1 can also be secreted in the non-acetylated form. In **Phase 2**, a feed-forward loop can be established by HMGB1 to enhance the inflammatory condition of the fetal cells. Depending on the concentration, HMGB1 mediates an inflammatory condition to neighboring cells. Higher concentrations of HMGB1 induce TLR2 and lower concentrations promote TLR4-mediated signaling resulting in p38MAPK activation mediated through activation of MAPK series of signaling molecules (MAPKKK →MAPKK →MAPK) (45). p38MAPK can lead to development of senescence phenotype (SP) in human fetal cells. Senescing cells cause a unique inflammatory signature that is known as “Senescence Associated Secretory Phenotype (SASP)”. With SASP, a unique set of proinflammatory cytokines, chemokines, growth factors, angiogenic factors and matrix degrading enzymes and their inhibitors may be activated independently through the p38MAPK pathway or with possible involvement of the NF-κB pathway (34; 35). In the absence of intraamniotic infection this sterile inflammation may produce similar outcomes as the well-defined infection-associated NF-κB pathway. Furthermore, with or without intraamniotic infection, this interaction may also activate HMGB1 and the autocrine effect of HMGB1 will continue the vicious cycle of inflammatory events until delivery. Inflammation seen at term, and in cases with PTB and pPROM, may arise from a senescence-oriented pathway mediated through generation of HMGB1 by oxidative stress or tissue injury.

Based on the data reported here, we propose a non-infectious activation inflammatory pathway in PTB and pPROM that can be mediated by various risk factors. Obesity, antioxidant deficient nutrition or malnourishment, behavioral risks (cigarette smoke, alcohol use, drug use etc.), and environmental pollutants that are common risk factors in both PTB and pPROM can generate oxidative stress and inflammation. In such conditions, membrane tissue injury causes HMGB1-mediated inflammatory response (Figure 6). We propose two phases of HMGB1 mechanisms in human fetal tissues that can potentially lead to uterotonic events. HMGB1 can provide a positive feedback loop enhancing fetal membrane senescence and related inflammation either as the acetylated form or even non acetylated molecule. Recent unpublished data from our laboratory also suggest that HMGB1 can be released from cells or they can also be packaged inside fetal cell derived exosomes (formed by multivesicular endosome fusion with the plasma membrane) and be transported to other tissues. This study also provides a mechanistic explanation for the role of HMGB1 in producing sterile inflammation as proposed by Romero et al [26].

The interaction of HMGB1 with senescence and inflammation needs further characterization in vitro and in animal models as the activation and activity of HMGB1 is likely dependent on the type and dose of stimulant. PTB and pPROM intervention strategies should consider HMGB1 as a potential mediator of this process.

We speculate that HMGB1-mediated senescence activation through the p38MAPK pathway may be a normal physiologic response promoting term labor. Fetal membranes and placenta experience considerable oxidative stress at term prior to initiation of labor. This can lead to tissue death, HMGB1 release and further enhancement of SP and SASP to promote labor at term. The inflammatory milieu observed at term is of non-infectious origin and HMGB1-mediated SP/SASP is likely one of the factors contributing to this phenomenon.
